# Divergent selection on home pen locomotor activity in a chicken model: Selection program, genetic parameters and direct response on activity and body weight

**DOI:** 10.1371/journal.pone.0182103

**Published:** 2017-08-10

**Authors:** Joergen B. Kjaer

**Affiliations:** Institute for Animal Welfare and Animal Husbandry, Friedrich-Loeffler-Institut, Celle, Germany; International Nutrition Inc, UNITED STATES

## Abstract

General locomotor activity (GLA) in poultry has attracted attention, as it negatively influences production costs (energy expenditure and feed consumption) and welfare parameters (bone strength, litter quality, feather pecking and cannibalism). Laying hen lines diverging in the average level of spontaneous locomotor activity in the home pen were developed by genetic selection using the founder New Hampshire line. Activity was recorded using RFID technology at around five weeks of age during four to five days in the home pen. After initial phenotyping, the least active birds were selected for the low activity line and the most active for the high activity line, with no gene transfer between lines. In each of six generations, approximately ten sires were mated to twenty dams producing 158 to 334 offspring per line per generation. The response to selection was rapid and of a considerable magnitude. In sixth generation, the level of GLA was approximately halved in the low and doubled in the high line compared to the control (7.2, 14.9 and 28.7 recordings/h). Estimated heritability of locomotor activity in the low and high line was 0.38 and 0.33, respectively. Males, in general, were more active than females. High line birds were significantly heavier than low line birds. In fourth, fifth, and sixth generation, low as well as high line birds were lighter than control line birds. This selection experiment demonstrates variation in heritability for GLA and, as a result, genetically diverged lines have been developed. These lines can be used as models for further studies of underlying physiological, neural and molecular genetic mechanisms of spontaneous locomotor activity.

## Introduction

General locomotor activity (GLA) has a wide phenotypic variation and a strong genetic background. While moderate levels of physical activity are associated with improved physical and mental health, extremely high levels of physical activity are associated with behavioural disorders such as attention deficit hyperactivity disorder (ADHD) [1]. In order to search for genetic and physiological mechanisms of GLA, rodent models have been developed [1]. However, the chicken as a genetic model in agriculture and medicine is gathering interest [2]. In poultry, GLA has attracted attention for a range of reasons. On the one hand, higher levels of GLA increase energy expenditure and feed consumption, as productivity traits might conflict with increased GLA [3–5]. On the other hand, a range of positive effects are expected, such as increased bone strength [6–8], increased bone density and reduced bending and twisting of bones [9]. Increased litter quality can also be expected in a more active flock, with a looser and drier litter. Maintaining access to loose litter helps reduce the level of damaging pecking behaviour such as feather pecking and cannibalism [10, 11]. Higher levels of GLA can also increase the use of the free range area, which can reduce the level of feather pecking [12] and increase nutrient intake from the pasture [13] which may positively affect meat quality parameters [14, 15].

Large differences in GLA exist between breeds. Broiler type chickens are usually less active than layer types [9, 16–18]. These differences even exist during the first days of life, where there is no substantial difference in body weight [19], and may be attributed to genetic differences in spontaneous locomotor activity. Differences have also been reported among layer strains [20, 21]. These large breed differences point to a substantial additive genetic component of GLA, but such estimates in chickens are lacking.

The present study focuses on home pen GLA in chickens, defined here as the ‘degree of movement’ of chickens during the whole light period in their home pen. This ‘movement’ was estimated by random sampling of chicken positions within the pen using individual transponders. The objectives of the study were to develop lines divergent in GLA through genetic selection, and to report direct and correlated responses to selection for GLA, growth rate and body weight.

## Materials and methods

Care and use of animals for the purpose in this study followed the guidelines of the European Communities Council Directive of 22 September 2010 (2010/63/EU) and the German Animal Protection Law. The experimental protocol with the number JK-02-2009 was reviewed and approved by the Institutional Animal Care and Use Committee (Colloquium) of FLI, Celle. All birds were housed and managed according to general farming procedures with the permit no. DE276033510060555.

### Lines and selection procedure in general

The selection experiment was initiated in 2009. The founder line was a New Hampshire laying line kept at the Institute. The selection study was initiated from a control line (S0) using a random mating scheme from 10 sires and 100 dams. A pedigree hatch of 456 chickens were produced from the control population. At 5 weeks all offspring were assessed for locomotor activity. The trait chosen for selection throughout the experiment was the mean number of activity bouts per h of recording during a light period of 11 h and a recording period of five consecutive days. Among the 456 chickens of the control line, the 20 females and 10 males with the lowest breeding values were selected as parents to produce the first selected generation (S1) of the low activity line. Similarly, the 20 females and 10 males with the highest breeding values were selected as parents to produce the first generation of the high activity line. From S1 and onwards the two experimental lines were kept as closed populations. The selection procedure is summarised in Table 1. Due to technical restrictions, no selection could be done in generation 5. The lines were reproduced from one male per sire and one female per dam chosen at random from the offspring. No results will be presented from this generation and the next selected generation is referred to as generation S5.

**Table 1:** Overview of the development of the selection lines.

Generation	Number of hatches	Line	Sires^¶^ per line, N	Dams^¶¶^per line, N	Offspring phenotyped per line, N
0 (founder)	1	Control	10	99	440
S1	2	Low	4	20	247
		High	5	19	294
S2	2	Low	10	19	318
		High	10	20	332
S3	2	Low	10	20	261
		High	10	20	309
S4	4	Low	10	20	297
		High	10	20	261
		Control	8	74	304
No selection	1	Low	10	20	0
		High	10	20	0
S5	1	Low	10	20	112
		High	10	20	120
		Control	4	30	120
S6	1	Low	10	20	166
		High	10	20	160
		Control	20	40	41

Control: Control line, New Hampshire line reproduced by random mating using 10 males and 100 females per generation.

Low: Line selected for low levels of locomotor activity in the home pen at 5 weeks of age.

High: Line selected for high levels of locomotor activity in the home pen at 5 weeks of age.

^¶^Sires are breeder males and

^¶¶^dams are breeder females.

### Housing and management

Chickens were hatched at the Institute and housed in a single mixed-sex and mixed-line group in a floor pen from hatch until 16 weeks of age. This home pen was in a climate controlled house (34°C lowered to 20°C during 5 weeks, 50–80% RH). Lighting program was 24L:0D for the first two days, 14L:10D for the next five days, 13L:11D for second week, 12L:12D for third week, 11.30L:12.30D for fourth week, 11L:13D for fifth week, 10.30L:13.30D for sixth week and 10L:14D for seventh to sixteenth week. Light was supplied only from overhead 60 W tungsten lamps to a light intensity of 30 to 70 lx at bird eye level. Pens were fitted with water-nipples and round feed troughs. Feed (main ingredients maize, wheat, barley and soya bean meal) and water was available *ad libitum*. A chicken starter (11.4 MJ ME/kg, 180 g/kg protein) was given from 0 to 7 weeks, a grower (11.3 MJ ME/kg, 144 g/kg protein) from 8 to 21 weeks and a layer feed (11.1 MJ ME/kg, 163 g/kg protein) from 22 weeks. A layer of 1–5 cm wood chips covered the floor. From 17 weeks of age, the birds were housed in floor pens with partial slatted floor and littered floor and with access to nests. S3 hens were housed in furnished cages from 17 weeks until reproduction. Reproduction was accomplished using artificial insemination and trap nests.

### General locomotor activity and body weight

**Fig 1:**
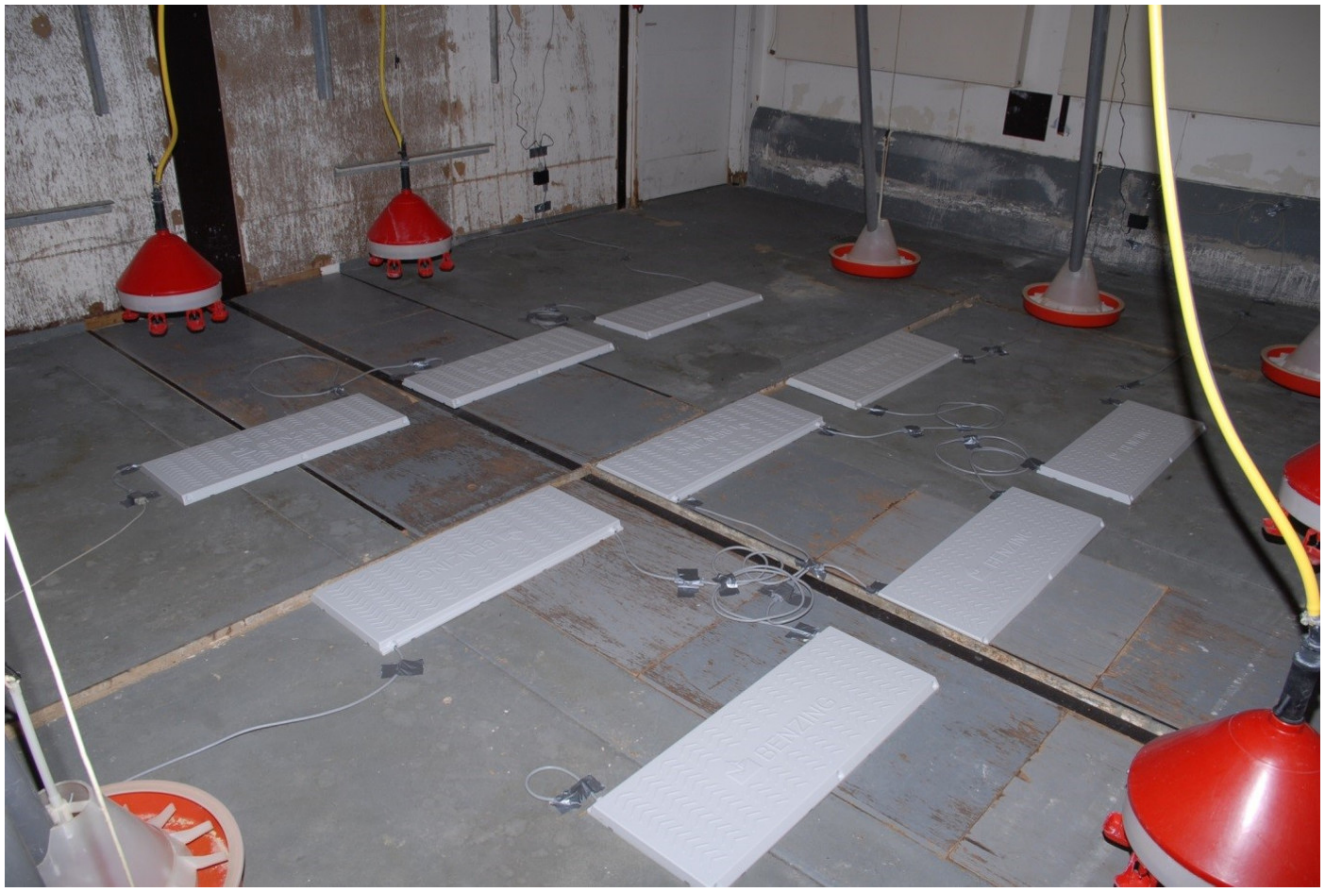
Experimental set up showing the placement of antennas covering the central part of the home pen. A 5 cm layer of cut straw was applied to the floor covering the antennas during the experiment. The recording period was the 11 h light period over five days totalling 55 h.

GLA is defined here as the ‘degree of movement’ of chickens during the whole light period as estimated by random sampling of chicken positions within the pen. GLA was recorded in the home pen using electronic transponders during a period of four to six days beginning at the age of 32 to 36 days, except for S0 beginning at 23 days of age. To avoid any bias from pen- or age-effects, birds from all lines and both sexes were recorded in the same pen, at the same time. They were fitted with a leg band carrying an electronic transponder (approx. 0.5 g, 2 mm in diameter and 10 mm long) emitting a unique number when close to an antenna. Nine sets of 12 circular antennas (each set 76 cm long and 30 cm wide, each single antenna with a diameter of 10 cm) were placed on the floor in a grid covering the central part of the littered pen (see Fig 1). The pen size was 28 m^2^ (6.1 m long and 4.6 m wide), of which the antennas covered 33%, equal to 9.3 m^2^. The system (Gantner Pigeon Systems GmbH, Schruns, Austria) scanned each antenna two times per second. When a transponder came within ca. 15 cm of an antenna, information for bird identity, antenna location, date and time of day was recorded. Scanning covered the whole 11 h lighting period each day and was done for five days (i.e. 55 h) of total recording time, except in first generation where it was four days (44 h). Results are presented as mean number of records per bird per hour. The birds were left alone, without behavioural restrictions of any kind during the recording period except for daily feeding and control routines, which lasted about 15 min per d.

Individual body weights (BW, ± 1 g) were recorded prior to activity recording at 5 weeks (32–36 days of age).

### Statistical analysis

Line and sex effects were, due to the unbalanced design, tested within generations using analysis of variance with individual records as statistical units. The initial model was: general locomotor activity (GLA) = Line + Sex + Line x Sex + Hatch + error, with errors assumed N (0,1). Presented data are in the original scale. The models were reduced appropriately according to results of significance testing. The response (GLA) was transformed using the natural logarithm or arc sin square root transformation as shown in the following. For first and sixth generation, the model was log (GLA) = Line + Gender + error, for second, third and fourth generation, it was log (activity) = Line + Gender + Hatch + error, and for fifth generation, it was arc sin (sqrt (GLA)/100) = Line + Gender + Line x Gender + error. Four birds that displayed extreme low activity were presumed not to have normal healthy behaviour, and were thus removed from the fifth generation’s dataset. The GLM-procedure (PROC GLM) in SAS 9.4 software was used for the calculations (SAS Institute Inc., Cary, IL). Breeding values for individual birds were estimated within lines and generations using family index [22]. The relationship matrix included all parents and offspring contributing to the particular line in the actual generation. The analysis was achieved using a restricted maximum likelihood (REML) procedure by means of the DMU package [23]. The model included fixed effect of sex and random effects of animal and sire. Data were standardised for hatch effects before calculations for generations S2, S3 and S4. Estimates of heritability and genetic correlations were based on the animal model procedure using the complete data set. Estimates of variance and covariance components were obtained from a REML analysis of a multi-trait mixed model using the DMU package. The models included activity and body weight as dependent variables, sex and generation as fixed effects, and animal and error as random effects. Phenotypic correlations were calculated as Pearson’s product-moment correlations within generations.

## Results

High line birds were more active than low line birds after just one generation of selection (Table 2). In fourth, fifth, and sixth generation, selection lines were compared to control line birds. Low line birds were less active and high line birds more active than control line birds in these three generations (Table 2).

**Table 2:** Locomotor activity^¶^ of New Hampshire chickens during six generations of divergent selection on locomotor activity in the home pen.

Gene-ration	N	Main effects									*R*^2^
		Line			Gender		*P*-value				
		Low^††^	Control^†^	High^†††^	Males	Females	Line	Gender	Line x Gender	Hatch	
0	440	-	12.1^1^ (0.24)	-	13.5^a^ (0.34)	10.6^b^ (0.31)	-	***	-	-	0.08
1	541	9.50^b^ (0.25)	-	14.6^a^ (0.31)	13.4^a^ (0.33)	10.7^b^ (0.30)	***	***	ns	ns	0.30
2	650	11.0^b^ (0.24)	-	18.4^a^ (0.38)	14.77 (0.35)	14.65 (0.43)	***	ns	ns	*	0.32
3	570	11.1^b^ (0.23)	-	26.4^a^ (0.45)	20.1^a^ (0.63)	17.5^b^ (0.53)	***	***	ns	***	0.62
4	862	7.26^c^ (0.20)	12.6^b^ (0.30)	25.8^a^ (0.62)	15.7^a^ (0.65)	14.7^b^ (0.40)	***	**	ns	***	0.60
5	346	6.40^c^ (0.32)	9.53^b^ (0.32)	16.70^a^ (0.66)	11.29 (0.60)	10.46 (0.42)	***	ns	*	-	0.49
6	367	7.16^c^ (0.33)	14.91^b^ (0.84)	28.70^a^ (0.64)	18.24^a^ (0.98)	15.62^b^ (0.78)	***	**	ns	-	0.74

^¶^Locomotor activity expressed as least squares means (SEM) of number of antennas passed per bird per h during four to six days of recording beginning at 32–36 days of age

^†^Control: Control line, New Hampshire line reproduced by random mating using 10 males and 100 females per generation.

^††^Low: Line selected for low levels of locomotor activity in the home pen at 5 weeks of age.

^†††^High: Line selected for high levels of locomotor activity in the home pen at 5 weeks of age.

N = number of observations; -: Not applicable.

^a, b, c^ Least Squares means with no common superscript letter differ significantly (*: *P* < 0.05; **: *P* < 0.01; ***: *P* < 0.001). ns: non-significant when *P* > 0.05.

^1^ Arithmetic mean.

*P*-values and *R*^2^ values are of the final model.

Males were more active than females in general, except in second and fifth generation (Table 2). In fifth generation, the Line x Gender interaction effect was significant (*P* < 0.05). Further analysis showed no significant gender effects within lines (*P* > 0.05), whereas line effects were very significant within gender (*P* < 0.0001).

The high line birds were significantly heavier than the low line birds in all selected generations (Table 3). In fourth, fifth and sixth generation, low as well as high line birds were lighter than control line birds (Table 3).

**Table 3:** Body weight of New Hampshire chickens during six generations of divergent selection on locomotor activity^¶^ in the home pen.

Gene- ration	N	Main effects									*R*^2^
		Line			Gender		*P*-value				
		Low^††^	Control^†^	High^†††^	Males	Females	Line	Gender	Line x Gender	Hatch	
0	440	-	289^1^ (2.01)	-	309^a^ (2.60)	270^b^ (2.44)	-	***	-	-	0.22
1	541	289^b^ (2.67)	-	303^a^ (2.76)	324^a^ (2.37)	268^b^ (2.01)	***	***	ns	ns	0.40
2	650	273^b^ (2.39)	-	299^a^ (2.34)	305^a^ (2.42)	267^b^ (1.94)	***	***	ns	***	0.29
3	570	331^b^ (2.89)	-	377^a^ (2.73)	383^a^ (2.90)	325^b^ (2.14)	***	***	**^2^	**	0.52
4	862	231^c^ (2.14)	253^a^ (2.73)	244^b^ (3.02)	263^a^ (2.83)	223^b^ (1.64)	***	***	ns	***	0.57
5	352	283^c^ (4.27)	363^a^ (3.60)	349^b^ (3.76)	359^a^ (4.04)	304^b^ (3.14)	***	***	ns	-	0.62
6	367	199^b^ (2.07)	233^a^ (4.63)	226^a^ (2.23)	228^a^ (2.37)	210^b^ (2.06)	***	***	ns	-	0.30

^¶^Locomotor activity expressed as least squares means (SEM) of number of antennas passed per bird per h during four to six days of recording beginning at 32–36 days of age

^†^Control: Control line, New Hampshire line reproduced by random mating using 10 males and 100 females per generation.

^††^Low: Line selected for low levels of locomotor activity in the home pen at 5 weeks of age.

^†††^High: Line selected for high levels of locomotor activity in the home pen at 5 weeks of age.

N = number of observations, -: Not applicable.

^a, b, c^ Least Squares means with no common superscript letter differ significantly (*: *P* < 0.05; **: *P* < 0.01; ***: *P* < 0.001).

ns: non-significant when *P* > 0.05.

^1^ Arithmetic mean.

^2^ The difference between males and females was a little larger (71 g) in the High line compared to the Low line (47 g), all four cross-effects being significantly different from each other (*P* < 0.01).

*P*-values and *R*^2^ values are of the final model.

Males were heavier than females in each generation (Table 3). There were low positive phenotypic correlations between GLA and body weight in all generations when using data from both lines (Table 4). When analysing within line, the correlations were generally lower and not different from zero in S2 and S5 (low line) and S2, S4 and S6 (high line).

**Table 4:** Phenotypic Pearson correlation coefficients between general locomotor activity and body weight at 35 days of New Hampshire chickens (both sexes combined) during six generations of divergent selection on locomotor activity in the home pen.

Gen	All			Low^†^ line			High^††^ line		
	N	r	*P*	N	r	*P*	N	r	*P*
S0	440	0.23	***	-	-	-	-	-	-
S1	541	0.34	***	247	0.24	***	294	0.34	***
S2	650	0.16	***	318	-0.02	ns	332	-0.01	ns
S3	570	0.45	***	261	0.25	***	309	0.21	***
S4	862	0.16	***	297	0.29	***	261	0.04	ns
S5	352	0.27	***	112	-0.04	ns	120	0.18	*
S6	367	0.41	***	166	0.19	*	160	0.13	ns

^†^Low line: Line selected for low levels of locomotor activity in the home pen at 5 weeks of age.

^††^High line: Line selected for high levels of locomotor activity in the home pen at 5 weeks of age.

N = number of observations, r- Phenotypic Pearson correlation coefficients

*: *P* < 0.05; **: *P* < 0.01; ***: *P* < 0.001; ns: non-significant when *P* > 0.05; -: Not applicable.

There was no gene transfer between lines after generation S0.

The estimated additive genetic variances for the low and high lines were 4.36 and 20.42, while the respective values for body weight were 779.5 and 455.8. The estimated values of phenotypic variances of GLA for the low and high lines were 11.56 and 61.40, while the respective values for body weight were 1459.5 and 1425.0.

Heritabilities and genetic correlations are shown in Table 5. A low to moderate heritability was found for GLA and a moderate heritability was found for BW. Genetic correlations between GLA and body weight were low.

**Table 5:** Heritability^¶^ of general locomotor activity, body weight at 35 days and genetic correlations in New Hampshire chickens during six generations of divergent selection on locomotor activity in the home pen.

Line	Heritability				Genetic correlation	
	Activity		body Weight		r_g Act-BW_	SE
	h^2^	SE	h^2^	SE		
Low^†^	0.38*	0.08	0.53*	0.06	0.15	0.14
High^††^	0.33*	0.06	0.32*	0.07	0.07	0.16

^¶^Heritability values are presented for combined data for all generations

^†^Low: Line selected for low levels of locomotor activity in the home pen at 5 weeks of age.

^††^High: Line selected for high levels of locomotor activity in the home pen at 5 weeks of age.

There was no gene transfer between the lines after generation S1.

h^2-^ heritability estimate; SE-Standard Error;

* Significantly different from zero (*P* < 0.05).

## Discussion

The response to selection for GLA was rapid and of a considerable magnitude. In sixth generation, the levels of GLA were approximately halved in the low and doubled in the high line compared to the control (7.2, 14.9 and 28.7 recordings/h), showing a fairly symmetric selection response. A report on selection for open-field activity in young chickens [24] presented a symmetric and quick response to selection and heritability estimates from 0.33 to 0.38, depending on variable recorded. High heritabilities were found in a similar selection study on open field behaviour in pheasants (*Phasianus colchicus*) [25]. Open-field activity differs from home pen locomotor activity due to factors such as fear and social reinstatement behaviour, as chickens are tested in isolation. It is therefore proposed that open-field behaviour in chickens represents a compromise between opposing tendencies, i.e. to reinstate contact with conspecifics and minimise detection in the face of possible predation [26]. Using a different approach, Bessei et al. [27] selected Japanese quail for five generations, which resulted in significant differentiation of the selected lines for their activity index. In their study, activity was recorded in single bird cages (25 cm x 25 cm) with a light beam crossing the cage and a photocell recording when a light beam was blocked by a moving bird.

In the present study, heritabilities of GLA were estimated to be 0.38 and 0.33 in the low and high lines, respectively. Jezierski and Bessei [21] recorded activity over two days in adult laying hens using single bird cages with a floor designed to record movement of birds within the cage (the ‘shuttle-box’), The two genotypes tested differed significantly in activity and the estimated activity-heritability of 0.18 was lower than the results found in the current study. A more recent study found low heritability (0.09 ± 0.07) of locomotor activity in 16 days old broiler chickens [28]. Unlike the current study's home pen and continuous eleven hour recording, Mignon-Grasteau [28] kept 10 days old chickens in individual cages and sampled behaviour (moving, feeding, lying) every 2 min during 1 h video recordings. This method might have given lower repeatability and thus lower heritability estimates. In rodent literature, home cage activity in mice was successfully selected during ten generations with a realised heritability of 0.40 [1]. Activity was recorded continuously over two days, in which mice were singly housed, though with limited access to other mice through a wire mesh. Another activity measure in rodents is voluntarily wheel running. This behaviour was also successfully selected yielding realised average heritability estimates of 0.19 [29].

In this study, the moderate heritability for body weight in the high line of 0.32 is comparable to the estimate of 0.25 found for body weight at 18 weeks in a New Hampshire line [30] as well as estimates of 9-week body weight for the Oklahoma Agricultural Experimental Station New Hampshire population [31]. The estimate in the low line (0.53) was more similar to those reviewed by Szwaczkowski [32] (0.32–0.68) in White Leghorn lines.

The current values for genetic correlation between GLA and body weight (0.07 and 0.15, not significantly different from zero) were lower than the estimate of 0.31 in chickens recorded in the shuttle-box [21]. In Japanese quail, Saleh and Bessei [33] found inconsistent correlations, negative in one line and positive in another line. In addition, an estimate of -0.21 was found in quail selected for dustbathing activity [34]. Majdak et al. [1] found a negative genetic correlation between activity and body weight in mice selected on increased home cage activity.

With small population sizes, the response to selection will usually underestimate the evolutionary potential of the original base population and may be confounded by drift and inbreeding. This is particularly important for interpreting correlated responses to selection. Rather than being due to pleiotropic effects of alleles favored by selection, they can reflect random fixation of alleles at loci unrelated to the targeted phenotype [35]. In the present experiment it is important to note that birds of both selected lines were lighter than control line birds in fourth, fifth and sixth generation, indicating that random fixation effects indeed might have occurred due to the relatively low population sizes.

The symmetric response gives no reason to believe that the trait selected for in the low line (sedentary behaviour) should be different from that selected for in the high line (activity). Emerging evidence suggests that physical activity and sedentary behaviour, reflected in physical inactivity, might be two different phenotypes that may have distinct underlying physiological mechanisms [36].

The motivational background for differences in locomotor activity is not well understood. Mice bred for increased activity (home cage or wheel running) have changes in the brain that appear to underlie increased motivation to run. The active lines respond entirely differently than controls to psychoactive drugs that increase dopamine signalling, such as amphetamine [1], apomorphine, cocaine, methylphenidate and GBR 12909 [35]. Only further genomic and neuro-physiological investigations can reveal if this applies in the present lines as well as in poultry in general.

## Conclusion

The selection experiments in this study successfully demonstrated heritable variation for general locomotor activity. The generated genetically diverged lines may be used as valuable models for further studies of underlying physiological, neural and molecular genetic mechanisms of spontaneous locomotor activity.
